# Correlation between maternal depression, anxiety, and stress and the children's oral health status and oral health-related quality of life: a cross-sectional study

**DOI:** 10.1007/s40368-025-01151-1

**Published:** 2026-01-10

**Authors:** Alaa Mohammed Yehia, Amira Saad Badran, Mahassen Mohamed Farghaly, Nagwa Mohammed Ali Khattab

**Affiliations:** 1 Pediatric Dentistry and Dental Public Health Department, Faculty of Dentistry, Ain Shams University, Cairo, Egypt; 2 Pediatric Dentistry and Dental Public Health Department, Faculty of Dentistry, Galala Univeristy, Suez, Egypt; 3 Pediatric Dentistry & Dental Public Health Department, Faculty of Oral & Dental Medicine, Misr International University, Cairo, Egypt

**Keywords:** Depression, Anxiety, Stress, Oral health-related quality of life, Oral hygiene, Correlation

## Abstract

**Purpose:**

The present study investigated how maternal depression, anxiety, and stress could affect the oral health status and oral health-related quality of life (OHRQoL) of preschool children.

**Methods:**

A total sample of 262 mother-children pairs were recruited. Maternal mental health status was assessed using the validated Arabic version of the Depression, Anxiety, and Stress Scale (DASS-21). The oral health status of preschool children aged 3 to 5 years was assessed using the dmft index and the debris index-simplified (DI-S). The children's OHRQoL was assessed using the validated Arabic version of the Early Childhood Oral Health Impact Scale (ECOHIS) questionnaire.

**Results:**

The mean (SD) depression scale score was 17.9 (9.9), whilst the mean (SD) anxiety scale score was 20.4 (10.7), and the mean (SD) stress scale score was 29.2 (8.3). There were statistically significant positive correlations between maternal depression and stress scores and their child's dmft scores. Similarly, there were statistically significant positive correlations between both maternal depression and anxiety scores, and their child's DI-S scores.

**Conclusions:**

Children of depressed or stressed mothers were more likely to have higher dmft scores. Similarly, children whose mothers had high levels of depression and anxiety had poorer oral hygiene. Maternal depression, anxiety, and stress had an inverse correlation with the OHRQoL of their children. Therefore, interventions that promote mothers’ mental health should be implemented at maternal and child health services, especially in those with severe and chronic forms of depression.

## Introduction

Mental health is defined as a state of mental well-being that enables people to cope with the stresses of life, realise their abilities, learn, work well, and contribute to their communities (World Health Organization 2021). Depression, anxiety, and stress are key dimensions of mental health; depression refers to persistent low mood and loss of interest, anxiety refers to excessive worry and tension, and stress refers to difficulty coping with external demands (GBD 2019 Mental Disorders Collaborators 2022).

Globally, mental disorders rank amongst the top 10 diseases, with anxiety and depression being the most prevalent (GBD 2019 Mental Disorders Collaborators 2022; Wu et al. 2023). Approximately 284 million people worldwide suffer from anxiety disorders, and 264 million people suffer from depressive disorders (World Health Organization 2017). The prevalence of maternal mental disorders amongst women in low and middle income countries is the highest at nearly 20% (McNab et al. 2022). In Egypt, the overall prevalence of mental disorders amongst adults aged 18 to 64 years was 16.93%, with women more than twice as likely as men to experience mental disorders (Ghanem et al. 2009).

Mental Health Screening Tools (MHSTs) are utilised to evaluate the mental health status of individuals and pinpoint potential signs or symptoms of psychological disorders, which warrant further evaluation, and aid clinicians in comprehending the conditions of individuals to inform therapeutic decisions (Shields et al. 2021). One of the widely utilised tools is the Depression, Anxiety, and Stress Scale (DASS-21) that is a self-reported questionnaire used to assess the severity of symptoms related to depression, anxiety and stress (Lovibond and Lovibond 1995; Antony et al. 1998; Ali et al. 2017).

Maternal mental disorders, particularly stress and depression, can negatively impact oral health behaviours both during pregnancy and after childbirth. Mothers experiencing high levels of stress or depression may engage in unhealthy behaviours, such as poor dietary habits, inadequate oral hygiene practices, and increased use of tobacco or alcohol, all of which can detrimentally affect their child's oral health (Sultana et al. 2023). These effects extend to children, influencing their development and well-being, particularly during the critical developmental period within their first five years-of-life (Mudiyanselage et al. 2024). Additionally, diminished maternal-infant bonding may disrupt attachment and hinder the socio-emotional development of their children (Le Bas et al. 2021).

Oral diseases remain a significant public health concern. Untreated carious lesions in primary teeth affect an estimated 510 million children globally (World Health Organization 2023). The Global Oral Health Status Report by WHO in 2019 indicated that the prevalence of caries in primary teeth in Egypt was 44.1% (World Health Organization 2023). Research has shown a link between dental plaque accumulation and the development of carious lesions as well as gingivitis (Davidovich et al. 2020). An evaluation of the oral health status of 6 year-old school children in Iran revealed marked differences in oral hygiene index-simplified (OHI-S) scores depending on how frequently they brushed their teeth and visited the dentist (Behbahani Rad et al. 2016). This suggests that encouraging good oral hygiene practices and provision of healthy diets with reduced sugar, especially in school environments, could enhance children's oral health outcomes (Behbahani Rad et al. 2016; Seni et al. 2025).

In addition to these clinical parameters, it is vital to evaluate the oral health-related quality of life (OHRQoL), as it reflects the individual's subjective assessment of their oral health, functional capabilities, emotional well-being, and self-perception (Sischo and Broder 2011), which can also be utilised to identify factors that contribute to a decline in OHRQoL, thus guiding oral healthcare priorities (Abanto et al. 2016; Moreira-Santos et al. 2023). The early childhood oral health impact scale (ECOHIS) is one of the most commonly used tools for assessing the impact of oral health problems and treatment on the daily lives of preschool-aged children (3–5 years) and their families (Pahel et al. 2007; Farsi et al. 2017).

To the best of the authors’ knowledge, no previous research has examined the correlation between maternal depression, anxiety, and stress and both the oral health status and OHRQoL of preschool children within our regional context. Given the growing recognition of maternal mental health as a key social determinant influencing child health outcomes, including oral health, there is a pressing need to better understand this relationship. The present study therefore aimed to investigate how maternal mental health is correlated with children’s oral health and OHRQoL. Identifying these relationships is important, as it can help inform preventive strategies and support interventions that promote both improved maternal mental health and enhanced oral health and quality of life among preschool children.

## Participants and methods

### Study design and ethical approval

This analytical cross-sectional study employed a survey and clinical examination to investigate the correlation between the mental health of mothers expressed as depression, anxiety, and stress, and the oral health, along with the OHRQoL of their preschool-aged children. Ethical approval for the study was granted by the Research Ethics Committee (FDASU-Rec), following the relevant guidelines and regulations of the Declaration of Helsinki, in January 2022, with the approval number (FDASU-Rec ID012222). A signed informed consent form in Arabic was acquired from mothers who agreed to participate in the study. The study protocol was also registered on https://clinicaltrials.gov/, assigned the ID number: NCT05753423.

### Sample size estimation and study participants

Using an alpha level of 0.05, a beta of 0.2 (indicating a power of 80%), and a correlation coefficient of -0.189 taken from a prior similar study (Gavic et al. 2018), the final calculated sample size was 262 mother–child dyads, after adding an additional 20% to offset potential non-responses and incomplete responses. The sample size calculation was performed using R statistical analysis software, version 4.1.2 for Windows 27 (R Core Team 2021). A convenience sample of Egyptian mothers with preschool children of both sexes, aged 3–5 years, who attended the paediatric dentistry outpatient clinic at the Faculty of Dentistry, Ain Shams University (FD-ASU), was recruited between September 2022 and February 2024. Only healthy children, without any long-term medications, physical, learning, or mental disabilities as indicated by their medical histories, were included.

### Data collection

The mental health of Egyptian mothers was evaluated using the validated Arabic version of the depression anxiety stress scale-21 (DASS-21) which is a quantitative measure that includes three subscales named depression (D), anxiety (A), and stress (S), with 7 items dedicated to each of these subscales (Lovibond and Lovibond 1995; Ali et al. 2017; Ali and Green 2017). Participants respond on a 4-point scale, ranging from 0 to 3, where 0 refers to “did not apply to me at all”, 1 refers to “applied to me to some degree, or some of the time”, 2 represents “applied to me to a considerable degree or a good part of the time” and 3 denotes “applied to me most of the time.” The total score for each subscale ranges from 0 to 21. Scores for the DASS-21 subscales are multiplied by 2 to derive the final scores, corresponding to those of the DASS-42. A higher total score for each subscale reflects an increased level of depression, anxiety, and/or stress. Scores below 9, 7, and 14 for depression, anxiety, and stress, respectively, are indicative of normal ranges.

The OHRQoL among preschool-aged children was assessed using the validated Arabic version of the ECOHIS questionnaire, which consists of 13 items divided into child and family impact sections (Pahel et al. 2007; Farsi et al. 2017). Response options were scored as follows: 0 = never, 1 = hardly ever, 2 = occasionally, 3 = often, 4 = very often, and 5 = do not know. The scoring for the child impact section ranges from 0 to 36, whilst the family impact section ranges from 0 to 16. Participants with over 2 missing responses in the child impact section or more than 1 missing response in the family impact section were excluded from the study.

All questionnaires were administered through interviewer-based interviews to accommodate participants with varying literacy levels. A single trained interviewer (the principal investigator) conducted all interviews to ensure consistency. Training was undertaken during the pilot phase and focused on reading each item clearly and neutrally, without interpretation or prompting, while maintaining standardised administration procedures. To minimise interviewer bias, particular care was taken to avoid verbal or non-verbal cues that might influence responses. To ensure anonymity and avoid social desirability bias, no personal identifiers such as names or National ID numbers were collected during data collection.

The oral health condition of the preschool children was evaluated by clinical examinations of both caries experience and oral hygiene status by a single trained examiner, who was also the principal investigator. Before starting data collection, the examiner underwent calibration and methodological training under the supervision of a senior professor in paediatric dentistry, who served as the reference standard. The calibration process included reviewing diagnostic criteria for dmft and the Debris Index–Simplified (DI-S), followed by joint clinical evaluation sessions to ensure consistency and accuracy in scoring. To assess intra-examiner reliability, the examiner recorded dmft and DI-S scores for 25 preschool children and repeated the examinations after a two-week interval. Reliability analysis demonstrated excellent agreement for the dmft scores, with an Intraclass Correlation Coefficient (ICC) of 0.92. For the DI-S, the results showed substantial agreement, with a weighted kappa coefficient of 0.86. The data collected from those children during training and calibration were not included in the study.

Caries lesions were assessed using the dmft (decayed, missed, and filled teeth) index for primary teeth, adhering to the guidelines set by the WHO (World Health Organization 2013; Pakkhesal et al. 2021). Additionally, oral hygiene was measured by the debris index-simplified (DI-S) component of the Oral Hygiene Index-Simplified (OHI-S), modified for primary teeth (Miglani et al. 1973; Behbahani Rad et al. 2016). The examined index teeth included the buccal surfaces of 55, 51, 65, and 71 and the lingual surfaces of 75 and 85; DI-S scores were calculated as the mean of examined surfaces; with higher scores indicated poorer oral cleanliness.

### Statistical analysis

All participant's responses were entered anonymously on Google Forms, accessed only via the principal investigator’s official email account, with access restricted to the authors to ensure confidentiality. Data were subsequently exported for analysis and numerical coding was applied where necessary. Categorical data were reported in terms of frequency and percentage, whereas numerical data were expressed as means and standard deviations (SD). Data distribution was assessed for normality using the Kolmogorov–Smirnov test, which indicated deviations from a normal distribution for some variables. However, given the relatively large sample size in this study (n = 262 mother–child pairs), the use of parametric tests was considered appropriate. According to the Central Limit Theorem, when sample size exceeds 30, parametric statistical tests are generally robust to violations of normality and tend to provide greater statistical power compared to non-parametric alternatives (Kwak and Kim 2017).

The ANOVA test was used to model the relationship between dependent variables (depression, anxiety, and stress) and each of the independent variables such as mother’s age, marital status, mother's educational level, mother's occupational status, and household income. Pearson correlation tests were used to analyse the relationships between maternal depression, anxiety, and stress and the children's oral health status and OHRQoL. A statistical significance threshold of p ≤ 0.05 was set for all tests, with the analysis conducted using IBM SPSS Statistics for Windows version 20 (IBM Corp., NY, USA).

## Results

In the research project, 262 mothers participated; almost half (51.1%) were between 30 and 39 years old, and the majority (93.9%) were married; other participants were either divorced or widowed. Over half of the mothers (58.8%) had education levels of secondary school or lower, whilst close to one-third possessed a university degree or higher. Furthermore, the majority of the mothers (82.4%) undertook home duties. Regarding household income, most mothers who participated (80.2%) had a monthly household income of less than 4000 Egyptian Pound (EGP).

As shown in Table 1, the mean (SD) depression scale score was 17.9 (9.9), whilst the mean (SD) anxiety scale score was 20.4 (10.7), and the mean (SD) stress scale score 29.2 (8.3).

**Table 1:** The mean values and the range of the depression, anxiety, and stress scale (DASS-21) amongst participating Egyptian mothers

Scale	Items of the DASS-21 scale	Mean (SD)	Range
Depression	I struggled to experience any positive feelings	1.7 (1)	(0–3)
	Taking the initiative to engage in activities felt challenging	1.6 (1.1)	(0–3)
	It seemed there was nothing ahead to anticipate	0.9 (1.2)	(0–3)
	I experienced sadness and distress	1.8 (1.1)	(0–3)
	I lost interest in everything	1.1 (1.1)	(0–3)
	I felt as though I had little worth as an individual	0.9 (1.2)	(0–3)
	I believed that life lacked purpose	0.9 (1.1)	(0–3)
Anxiety	I experienced dryness in my throat	1.2 (1.2)	(0–3)
	I had trouble breathing, with symptoms like rapid panting not linked to physical exertion	1.4 (1.2)	(0–3)
	I noticed a tremor in my hands, for instance	1.5 (1.2)	(0–3)
	I was anxious about situations where I might lose control and embarrass myself	1.8 (1.2)	(0–3)
	I felt as if I was on the verge of an unexpected wave of fear	1.4 (1.2)	(0–3)
	I could feel my heart racing without engaging in any physical activity	1.5 (1.2)	(0–3)
	I experienced fear without any clear reason	1.4 (1.2)	(0–3)
Stress	I struggled to find relaxation and peace	2.1 (1)	(0–3)
	I often overreact to situations and occurrences	2.3 (1)	(0–3)
	I felt that I was expending a significant amount of nervous energy, affecting my ability to handle stress	2.6 (0.7)	(0–3)
	I experienced feelings of disturbance and agitation	1.8 (1)	(0–3)
	I have a hard time feeling at ease	2 (1)	(0–3)
	I couldn't tolerate anything that obstructed my desires	1.6 (1.2)	(0–3)
	I noticed that I tended to become angry rapidly	2.1 (1.1)	(0–3)
Total scores	Depression scale	17.9 (9.9)	(0–42)
	Anxiety scale	20.4 (10.7)	(0–42)
	Stress scale	29.2 (8.3)	(2–42)

Table 2 shows that the mean depression scores were the highest amongst the 30-to-39-year age group, widowed, illiterate, non-employed mothers, and those whose monthly household income was less than 2000 EGP. Similarly, the mean anxiety and stress scores were the highest amongst 30-to-39-year age group, divorced, illiterate, those with less than secondary education, non-employed mothers, and those whose monthly household income was less than 2000 EGP.

**Table 2:** Mean (SD) of maternal depression, anxiety, and stress values for participants with different socio-demographic characteristics

Socio-demographic characteristics	Mothers' responses	Depression Mean (SD)	Anxiety Mean (SD)	Stress Mean (SD)
Mother's age^†^ (years)	20–29	17.3 (10.2)	20.7 (10.5)	28.1 (8.2)
	30–39	18.6 (9.4)	20.7 (10.6)	29.9 (8.3)
	40–49	16.5 (11.3)	18 (11.5)	29.4 (7.8)
P-value		0.361, η^2^ = 0.007	0.304, η^2^ = 0.007	0.210, η^2^ = 0.010
Marital status^†^	Married	17.7 (9.8)	20.3 (10.8)	29.1 (8.3)
	Widow	23 (14.4)	20.5 (8.3)	30.5 (8.7)
	Divorced	20.3 (10.8)	22.1 (7.9)	31.2 (7.3)
P-value		0.356, η^2^ = 0.006	0.809, η^2^ = 0.001	0.584, η^2^ = 0.003
Mother’s educational level^†^	Illiterate	23.8 (12)	22 (10.4)	30.9 (8.7)
	Secondary or less	18.7 (9.8)	21.6 (10.8)	29.9 (7.9)
	University or more	15 (8.7)	17.8 (10.3)	27.6 (8.5)
P-value		< 0.001*, η^2^ = 0.061	0.011*, η^2^ = 0.029	0.05*, η^2^ = 0.020
Mother’s occupational status^‡^	Home duties	18.1 (9.8)	20.9 (10.7)	29.4 (8.3)
	Working	16.8 (10.4)	18.1 (10.4)	28.1 (8.1)
P-value		0.400, η^2^ = 0.003	0.082, η^2^ = 0.010	0.314, η^2^ = 0.004
Household income^†^	< 2000 EGP	22.5 (10.1)	23.7 (10.7)	31.3 (7.1)
	2001–4000 EGP	17.1 (9.6)	19.9 (10.4)	28.6 (8.5)
	4001–6000 EGP	14.8 (9.3)	19 (11.4)	28.3 (8.7)
	> 6000 EGP	11.7 (4.6)	13.8 (7.6)	27.1 (7.7)
P-value		< 0.001*, η^2^ = 0.096	0.001*, η^2^ = 0.051	0.05*, η^2^ = 0.026

(*) P-value ≤ 0.05

^**†**^One-way ANOVA was used

^**‡**^Independent samples t-test was used

η^2^ = effect size (eta squared)

Variations in the mean scores across maternal age, marital status, and occupational status had very small effect sizes (η^2^ < 0.01) indicating that these differences were of a little clinical significance. In contrast, maternal educational level and household income demonstrated statistically significant differences in depression, anxiety, and stress scores, with small to moderate effect sizes (η^2^ ranging from 0.02 to 0.096). Mothers with university-level education or higher and those with higher household income consistently showed lower levels of depression, anxiety, and stress.

When assessing the oral health status of the preschool children, the mean (SD) dmft score was 7.5 (4.2) and the mean (SD) value of the debris component (DI-S) of the oral hygiene index-simplified (OHI-S) was 1.1 (0.5).

Table 3 shows that the mean (SD) OHRQoL score of preschool children (as reported by their mothers) was 18.9 (8.9). It was also noted that the oral health status of the children impacted their quality of life more than its impact on their families where the child impact score had a mean (SD) of 11.2 (6.2), whilst the family impact score had a mean (SD) of 7.7 (3.6).

**Table 3:** The mean values and the range of the children's OHRQoL using the Early Childhood Oral Health Impact Scale (ECOHIS)

ECOHIS sections	Mean (SD)	Range
Child Impact section	11.2 (6.2)	(0–26)
Family Impact section	7.7 (3.6)	(0–16)
Total ECOHIS scale score	18.9 (8.9)	(0–41)

As reported in Table 4, there was a statistically significant weak positive correlation between maternal depression and stress scores and the dmft score of their preschool children. Similarly, there was a statistically significant weak positive correlation between both depression and anxiety scores and the total Debris score of the children (DI-S). This indicates that worse mental health status amongst the participating Egyptian mothers was associated with higher caries experience and worse oral hygiene status amongst their preschool children.

**Table 4:** Correlation between maternal depression, anxiety, and stress and the oral health status of their children expressed as dmft, Debris score (DI-S)

Oral health status		Depression scale	Anxiety scale	Stress scale
dmft	r	0.165**	0.035	0.130*
	P-value	0.003	0.537	0.021
Debris score of the children (DI-S)	r	0.117*	0.111*	0.084
	P-value	0.038	0.049	0.139

(*) P-value ≤ 0.05.

(**) P-value ≤ 0.005.

(r) Pearson correlation test was used.

A statistically significant weak positive correlation was also found between maternal depression, anxiety, and stress and the OHRQoL scale score of their children, which means that worse maternal mental health status, expressed as depression, anxiety, or stress, is associated with impaired quality of life amongst the children (Table 5).

**Table 5:** Correlation between maternal depression, anxiety, and stress and the OHRQoL of their preschool children

OHRQoL of children		Depression scale	Anxiety scale	Stress scale
Total child section	r	0.301^**^	0.288^**^	0.293^**^
	P-value	< 0.001*	< 0.001*	< 0.001*
Total family section	r	0.264^**^	0.271^**^	0.289^**^
	P-value	< 0.001*	< 0.001*	< 0.001*
Total OHRQoL scale score	r	0.316^**^	0.309^**^	0.320^**^
	P-value	< 0.001*	< 0.001*	< 0.001*

(*) P-value ≤ 0.05

(**) P-value ≤ 0.005

(r) Pearson correlation test was used

## Discussion

To the best of the authors’ knowledge, the present study represents the first investigation to explore the correlation between maternal depression, anxiety, and stress, assessed using the DASS-21, and the oral health status and OHRQoL of preschool children in Egypt. The study was undertaken to test a potential correlation that may provide evidence for integrating maternal mental health into the maternal-child care framework (Rahman et al. 2013). The DASS-21 was selected because it is applicable in both clinical settings for diagnostic and outcome monitoring and in non-clinical settings as a screening tool (Lovibond and Lovibond 1995; Antony et al. 1998).

In the present study, the mean maternal depression, anxiety, and stress scores were different amongst the different socio-demographic characteristics. The findings that higher mean depression, anxiety, and stress scores were found amongst mothers less than 39 years than in older ones are consistent with previous research, which revealed that younger age groups were more vulnerable to stress, depression, and anxiety symptoms (Varma et al. 2021; Aldhahir 2024). These findings can be explained as older individuals usually report higher levels of resilient coping, which may serve as a critical factor in safeguarding against psychological distress. In addition, young adults are often more vulnerable to financial distress due to increased financial responsibilities and the likelihood of having young children (Varma et al. 2021).

For the maternal educational level, the finding that illiterate individuals and those with less than secondary education have significantly higher mean depression, anxiety, and stress scores is in line with the results of previous research where women with a lower level of education had a higher risk of anxiety-depressive state (Collier Villaume et al. 2023; Joannès et al. 2023). This association may be attributable to the challenges faced by individuals with lower educational levels, who often encounter economic hardship and stress due to their limited access to favourable employment opportunities, which subsequently affects their mental health (Ross and Wu 1995).

The finding that non-working mothers have higher depression, anxiety and stress mean scores is consistent with previous findings which concluded that working mothers may have better health, fewer depressive symptoms and higher self-esteem compared to women only undertaking home duties (Buehler and O’Brien 2011; Melissa et al. 2015; D’arqom et al. 2021). In contrast, a study involving Indonesian women reported that working females were more susceptible to anxiety symptoms than women only undertaking home duties, suggesting a potential conflict in findings, putatively due to this study not using a validated and standardised anxiety measurement (Megatsari et al. 2020). In the present study, the association between maternal working status and their mental health was not statistically significant; however, it can give an explanation for the statistically significant association between household income and maternal mental health, where families with working mothers would probably have higher household income than those with non-working mothers.

The mean dmft score amongst preschool children in this study was higher than those reported in two comparable studies (Shalan and Abobakr 2018; Elfagi et al. 2021); however, it was comparable to the findings of other investigations that reported similar mean dmft scores (Adil et al. 2020; Ismail 2022). For assessing the oral hygiene of preschool children, the mean DI-S score (1.1) in the present study was found to be within the range of fair oral hygiene (0.7–1.8), which is consistent with those of previous studies (Shaghaghian et al. 2015; Osadolor and Iwuoha 2019). On the other hand, it was higher than that of 6 year-old schoolchildren in Iran (Behbahani Rad et al. 2016). The variation in mean dmft and DI-S scores can be explained partially by the different recruitment settings in which children in the present study and similar studies with higher mean dmft and DI-S scores were recruited from dental clinics or hospitals, in contrast to studies with lower mean scores that recruited children from kindergartens.

The present study showed a statistically significant positive correlation between maternal depression and stress and the dmft scores of their children. Similarly, maternal depression and anxiety had a significant positive correlation with children's debris scores. These findings are in accordance with previous studies that reported a significant association between parental stress and early childhood caries (Tang et al. 2005; Menon et al. 2013). Potential underlying mechanisms may involve multiple pathways. From a behavioural perspective, poor maternal mental health may contribute to inconsistent supervision of children’s oral hygiene and irregular dental attendance. From a biological perspective, altered inflammatory responses associated with maternal stress may affect the child’s oral environment and increase susceptibility to dental caries. From a social perspective, limited access to oral health services, healthy diets, and preventive care may further exacerbate oral health challenges. All of these factors can adversely affect children’s oral health status (Gomes et al. 2020; Tiwari et al. 2022; Adeniyi et al. 2025).

In the present study, it was noted that the mean ECOHIS score of the participating children was similar to those in studies conducted in other countries (Shaghaghian et al. 2015; Abanto et al. 2016; Yawary et al. 2016; Yang et al. 2023), but higher than two other studies conducted in Hong Kong (Wong et al. 2011; Jiang et al. 2019). Similar to what was shown for dmft scores, the difference in the mean OHRQoL scores could be explained by the fact that children in the present study and similar studies were recruited from dental clinics or hospitals where association was observed between poorer oral health in children seeking dental treatment and lower reported OHRQoL, whilst those in the studies with lower mean dmft scores were from kindergartens.

Consistent with a previous study, it was emphasised that maternal depression, anxiety, and stress have significantly positive correlations with their child’s OHRQoL scores (Costa et al. 2017). Potential reasons for such a correlation could be that mothers' neglect of their children’s oral hygiene and/or treatment needs could impair their oral health condition, and negatively impact their OHRQoL (Costa et al. 2017). In addition, children of mothers with depression or anxiety may exhibit altered dietary habits, such as increased consumption of cariogenic foods or irregular eating patterns, further affecting oral health outcomes (Lazarus et al. 2026). Moreover, mothers experiencing depression and anxiety are more likely to identify negative emotions whilst expressing their perceptions regarding their children’s OHRQoL (Goettems et al. 2011).

One of the main strengths of the present study is the use of validated Arabic versions of all applied questionnaires. This ensures standardisation of the methodology and that the findings can be easily compared with previous studies used the same validated questionnaires. However, certain limitations must be acknowledged. The cross-sectional design restricts the ability to infer causality, limiting the findings to associations and correlations. Furthermore, the convenience sampling method utilised may affect the generalisability of the results, despite its practicality when dealing with a diverse group such as Egyptian mothers (Mohsin Hassan Alvi 2016). The assessment of maternal mental health and children’s OHRQoL relied on self-reported measures, which may be subject to social desirability, however, this was minimised by ensuring anonymity of the participants. Furthermore, the analyses did not include multiple regression to adjust for potential confounders, so some associations may have been partially influenced by underlying differences in sociodemographic characteristics. Therefore, future studies are recommended to use longitudinal designs and multivariable regression models to ascertain causation and better clarify the direction and independence of these relationships.

## Conclusions

Socio-demographic characteristics of Egyptian mothers, such as high educational level and high household income, were found to have a significant positive impact on their mental health in terms of depression, anxiety, and stress. Higher levels of maternal depression and stress were associated with increased dmft scores in their children, indicating greater caries experience. Similarly, higher maternal depression and anxiety scores were correlated with higher DI-S scores, reflecting poorer oral hygiene in the children. In addition, higher maternal depression, anxiety, and stress were associated with lower OHRQoL scores in their children, demonstrating a negative impact on perceived oral health-related quality of life. Therefore, interventions that promote mothers’ mental health should be implemented at maternal and child health services, especially in those with severe and chronic forms of depression.

## Data Availability

No datasets were generated or analysed during the current study.
